# Identification of mitochondria-related feature genes for predicting type 2 diabetes mellitus using machine learning methods

**DOI:** 10.3389/fendo.2025.1501159

**Published:** 2025-03-27

**Authors:** Xiuping Xuan, Mingjin Sun, Donghui Hu, Chunli Lu

**Affiliations:** ^1^ Department of Endocrinology, The First Affiliated Hospital of Guangxi Medical University, Nanning, Guangxi, China; ^2^ Department of Endocrinology, Suizhou Hospital, Hubei University of Medicine, Suizhou, Hubei, China; ^3^ Department of Reproduction and Infertility, Suizhou Hospital, Hubei University of Medicine, Suizhou, Hubei, China

**Keywords:** type 2 diabetes mellitus, mitochondria, immune cells, machine learning, MR

## Abstract

**Purpose:**

We aimed to identify the mitochondria-related feature genes associated with type 2 diabetes mellitus and explore their potential roles in immune cell infiltration.

**Methods:**

Datasets from GSE41762, GSE38642, GSE25724, and GSE20966 were obtained from the Gene Expression Omnibus database. Weighted Gene Co-expression Network Analysis was performed to achieve mitochondria-related hub genes. Random Forest, Least Absolute Shrinkage and Selection Operator, and Support Vector Machines-Recursive Feature Elimination algorithms were used to screen mitochondria-related feature genes. Receiver Operating Characteristic analysis was applied to evaluate the accuracy of the feature genes. Pearson’s correlation analysis was used to calculate the correlations between feature genes and immune cell infiltration. The prediction of candidate drugs targeting the feature genes were predicted using the DGIdb database. qRT-PCR was performed to access the mRNA expressions of the feature genes.

**Results:**

Five mitochondria-related feature genes (SLC2A2, ENTPD3, ARG2, CHL1, and RASGRP1) were identified for type 2 diabetes mellitus prediction. They possessed high predictive accuracies with the area under the Receiver Operating Characteristic curve values >0.8. All five genes showed the strongest positive correlation with regulatory T cells and negative correlation with neutrophils. Additionally, drugs prediction analysis revealed 2(S)-amino-6-boronohexanoic acid, difluoromethylornithine, and compound 9 could target ARG2, while metformin was a candidate drug for SCL2A2. Finally, all five genes were confirmed to be decreased in MIN6 cells treated with high glucose and palmitic acid.

**Conclusion:**

SLC2A2, ENTPD3, ARG2, CHL1, and RASGRP1 could be used as the mitochondria-related feature genes to predict type 2 diabetes mellitus and the therapeutic targets.

## 1 Introduction

Type 2 diabetes mellitus (T2DM) is a metabolic disease, which is prevalent worldwide and characterized by upregulated blood glucose concentration (1). In 2021, 536.6 million individuals are estimated to live with T2DM, and the number is still rising (2). T2DM will lead to hyperglycemia and other life-threatening complications, including diabetic retinopathy and diabetic nephropathy (3). It accounts for approximately 90%–95% of all diabetes cases and is associated with significant morbidity and mortality due to these complications (4). Currently, lifestyle management and hypoglycemic drugs such as metformin and glucagon-like peptide-1 (GLP1) receptor agonists are effective therapeutic approaches for T2DM. However, long-term use of these drugs will result in side effects (5). Hence, there is an still urgent need to unveil the potential therapeutic targets for T2DM.

Mitochondria, the critical organelle for oxidative phosphorylation, fatty acid oxidation, cellular energy metabolism, and reactive oxygen species (ROS) production, is closely related to T2DM development (6, 7). High glucose, the primary feature of T2DM, can trigger elevated production of ROS. ROS will further damage the function of mitochondria, which finally causes decreased insulin secretion and the apoptosis of pancreatic β-cells (8). Eliminating ROS can attenuate insulin resistance, which is the primary cause of T2DM (9). Furthermore, deficiency in fatty acid metabolism of mitochondria results in insulin resistance (10). In addition to ROS production, the dysfunction of mitochondria will cause inflammatory response and promote the secretion of inflammatory factors, which will eventually lead to immune cell infiltration into the injured tissue, such as lymphocyte (11), macrophage (12), and neutrophil (13). The infiltration of immune cells will further aggravate the development of T2DM resulting in insulin resistance and β-cells dysfunction (14). However, whether any mitochondria-related genes can serve as candidate predictive genes for T2DM and whether they are associated with immune cell infiltration is unclear.

With the development of bioinformatics technology, the researchers can explore the critical risks or genes for disease prediction. A previous study revealed that three miRNAs could act as diagnostic targets for T2DM-related periodontitis via machine learning (15). In addition, machine learning indicated that bile acid, ceramide, amino acid, and hexose were risk factors for T2DM development (16). Eight ferroptosis-related genes in T2DM were also identified by machine learning (17). Several machine learning algorithms, including Support Vector Machines-Recursive Feature Elimination (SVM-RFE), Least Absolute Shrinkage and Selection Operator (LASSO) regression, Random Forest (RF) (18), Artificial Neural Network (19), and extended odd Weibull Rayleigh (20) were used to identify the feature genes or establish the predictive model for different diseases. In this study, we aimed to investigate the mitochondria-related feature genes for T2DM prediction and therapy by machine learning. Meanwhile, we further explored the relationships of the mitochondria-related genes and immune cells infiltration. Our study uncovered novel biomarkers and therapeutic targets related to mitochondria for T2DM. While mitochondrial dysfunction has been implicated in T2DM pathogenesis, the specific mitochondrial-related genes driving disease progression and their potential as predictive biomarkers remained poorly understood. Furthermore, the interplay between mitochondrial genes and immune cell infiltration in T2DM has not been systematically explored. This study aimed to fill these critical gaps in the literature by identifying key mitochondria-related genes predictive of T2DM and uncovering their relationships with immune cell dynamics, thereby providing new insights for personalized prediction and targeted therapy.

## 2 Methods and materials

### 2.1 Data collection and processing

The transcriptional profiles of pancreatic islets from patients with or without T2DM were obtained from the Gene Expression Omnibus (GEO) datasets (GSE41762, GSE38642, GSE25724, and GSE20966) (**Table 1**). Among them, the datasets of GSE41762, GSE38642, and GSE25724 were used as the training sets. The dataset of GSE20966 was employed as the validation set. When processing the data, we first matched the probes in each dataset to the corresponding gene name and deleted the empty probes. For those multiple probes that corresponded to the same gene, the mid-value of the probe-levels was used as the gene expression. Datasets were normalized using normalizeBetweenArrays in R language. After data integration of GSE41762, GSE38642, and GSE25724, ComBat of sva package was conducted to remove the batch effect. In addition, the 1,136 mitochondrial genes were downloaded from MitoCarta3.0 (https://www.broadinstitute.org/mitocarta/mitocarta30-inventory-mammalian-mitochondrial-proteins-and-pathways).

**Table 1:** Sample information in each dataset.

ID	Platform	Sample	Sample size	Data type
GSE41762	GPL6244	Pancreatic islets from patients with or without T2DM	77(20:57)	mRNA array
GSE38642	GPL6244	Pancreatic islets from patients with or without T2DM	63(9:54)	mRNA array
GSE25724	GPL96	Pancreatic islets from patients with or without T2DM	13(6:7)	mRNA array
GSE20966	GPL1352	Beta-cells from pancreases of patients with or without T2DM	20(10:10)	mRNA array

### 2.2 Different expression genes 

The “limma” package in R language was employed to obtain DEGs between disease and control samples in the merged dataset by setting the criteria as |logFC| > 0.3 and *p* value < 0.05.

### 2.3 Functional enrichment analyses of DEGs

The “clusterProfiler” package in R language was used to carry out the gene ontology (GO) and Kyoto Encyclopedia of Genes and Genomes (KEGG) enrichment analysis. The *p*-value was adjusted by Benjamini–Hochberg; then, the adjusted *p* value was used to display the results of the functional enrichment analyses of DEGs.

### 2.4 Enrichment scores of mitochondrial related DEGs

The mitochondrial-related DEGs in pancreatic islets from patients with or without T2DM were obtained by intersecting the DEGs and the 1,136 mitochondrial genes. Subsequently, the single-sample gene set enrichment analysis (ssGSEA) enrichment scores of mitochondrial-related DEGs were analyzed using the “gene set variation analysis (GSVA)” package of R language.

### 2.5 Weighted Gene Co-expression Network Analysis analysis

“WGCNA” package of R language was used to establish the gene expression modules specific to mitochondria according to the enrichment scores of the mitochondrial-related DEGs. We selected the DEGs in the top 50% of variance in the merged dataset as the input data. The optimal soft threshold was determined based on the scale-free topology criterion. Then, the weighted adjacency matrix was transformed to topological overlap matrix. The modules with more than 30 genes were screened by hierarchical clustering. The consensus modules were combined by setting the distance to 0.25. Each module was displayed in a random color. Then, the modules were screened based on phenotypic correlation. Mitochondria-related hub genes were identified based on module membership (MM, |MM| > 0.6) and gene significance (GS, |GS| > 0.5). The intersection of DEGs and the mitochondria-related hub genes was employed for further analysis.

### 2.6 Feature genes identification

To identify the feature genes associated with mitochondria in T2DM, we first utilized SVM-RFE to remove the redundant factors via the SVM function in “caret” package of R language. Then, functions = caretFuncs was specified in rfeControl, and method = “svmRadial” in rfe to predict the optimal features. LASSO regression was conducted using the “glmnet” package in R language to calculate and select linear models and retain valuable variables. Then, binomial distribution variables were used for LASSO classification, and the optimal variables were selected by choosing lambda 1se as the criterion. Subsequently, RF was employed to sequence genes, and the genes with Gini coefficient >1 were considered as important feature genes. The intersection of genes obtained by the three algorithms was defined as the final feature genes. The R package “pROC” was used to display the diagnostic performance of feature genes in different datasets.

### 2.7 Nomogram construction and evaluation

“rms” package in R language was used to construct the nomogram predicted model according to the risk factors, and the nomogram was visualized by using the “regplot” package in R language. Then, the clinical applicability of the nomogram was evaluated using calibration curves via calibrate function in “rms” package and decision curve analysis (DCA) via “ggDCA” package in R language.

### 2.8 Protein–protein interaction network

The candidate genes that would bind to the feature genes were analyzed by GeneMANIA (http://www.genemania.org).

### 2.9 Immune cell infiltration analysis

The ssGSEA enrichment scores of 28 immune cell subtypes were analyzed using the “GSVA” package of R language. The enrichment of different immune cell subtypes in different samples was obtained by transforming the expression matrix of genes between different samples into the expression matrix of gene sets between samples. Wilcox.test was applied to assess the difference in immune cell abundance between groups. Pearson’s correlation analysis was used to calculate the correlations between feature genes and immune cells.

### 2.10 Identification of candidate drugs

The Drug–Gene Interaction Database (DGIdb, www.dgidb.org) was used to explore the candidate drugs that would target the feature genes. The interaction network was visualized by Cytoscape software.

### 2.11 Cell culture

MIN6 cells were purchased from ATCC and cultured in Dulbecco’s modified Eagle’s medium (DMEM) medium with 12% FBS in an incubator with 5% CO_2_ at 37°C. The cells were divided into two groups: CON group [5 mmol/L glucose + bovine serum albumin (BSA)] and T2DM group [25 mmol/L glucose + 0.5 mmol/L palmitic acid (PA) + BSA]. Then, the cells were harvested at 48 h post-treatment.

### 2.12 Quantitative reverse transcription polymerase chain reaction

Total RNA was extracted by TRIpure (EP013, ELK Biotechnology, Wuhan, China). An equal amount of RNA was reverse transcribed into cDNA using the EntiLink™ 1st Strand cDNA Synthesis Super Mix (EQ031, ELK Biotechnology, Wuhan, China). The qRT-PCR amplification was performed in the QuantStudio 6 Flex System PCR instrument (Life technologies, San Diego, CA) by using the EnTurbo™ SYBR Green PCR SuperMix kit (EQ001, ELK Biotechnology, Wuhan, China). GAPDH was used as internal reference. The primer sequences used for qRT-PCR are listed in **Table 2**.

**Table 2:** The primer sequences.

Name	Forward (5′–3′)	Reverse (3′–5′)
GAPDH	TGAAGGGTGGAGCCAAAAG	AGTCTTCTGGGTGGCAGTGAT
SLC2A2	TGTCAGAAGACAAGATCACCGG	CTCTTGAGGTGCATTGATCACAC
ENTPD3	GACTTCTGCAGACACACTTGGAG	GTATCCATTTACGAGCAAGTGGT
ARG2	TCTGGTTGTGTATCCTCGTTCAG	GTATTAATGTCCGCATGAGCATC
CHL1	AGAATATGCTGGCTTATATGATGAC	CCTCTTCACAAAGCAAATAGTTAAC
RASGRP1	CGACACGACCCAAATTAATTC	GACAGTTCTTCAGGTTCCAGATG

### 2.13 Statistical analysis

All statistical analyses were performed using R language (v4.3.0). “FactoMineR” and “factoextra” packages were used for principal component analysis (PCA) and its visualization. Heatmap was visualized using the “pheatmap” package. The “ggvenn” package and “pROC” package were applied for Venn diagram and ROC curve visualization, respectively. “Corrplot” package was used for feature gene correlation analysis and its visualization. The “RCircos” package was employed to show the locations of genes on chromosomes. “Ggplot2” or “plot” was used to plot other results. The correlations between feature genes and immune cells were conducted by Pearson’s correlation analysis. Wilcox.test was used to test the difference in immune cell abundance between groups. *p* < 0.05 indicated a statistical difference. *p < 0.05, **p < 0.01, ***p < 0.001, ns meant no statistical difference.

## 3 Results

### 3.1 Identification of the DEGs in pancreatic islets from patients with or without T2DM

**Figure 1:**
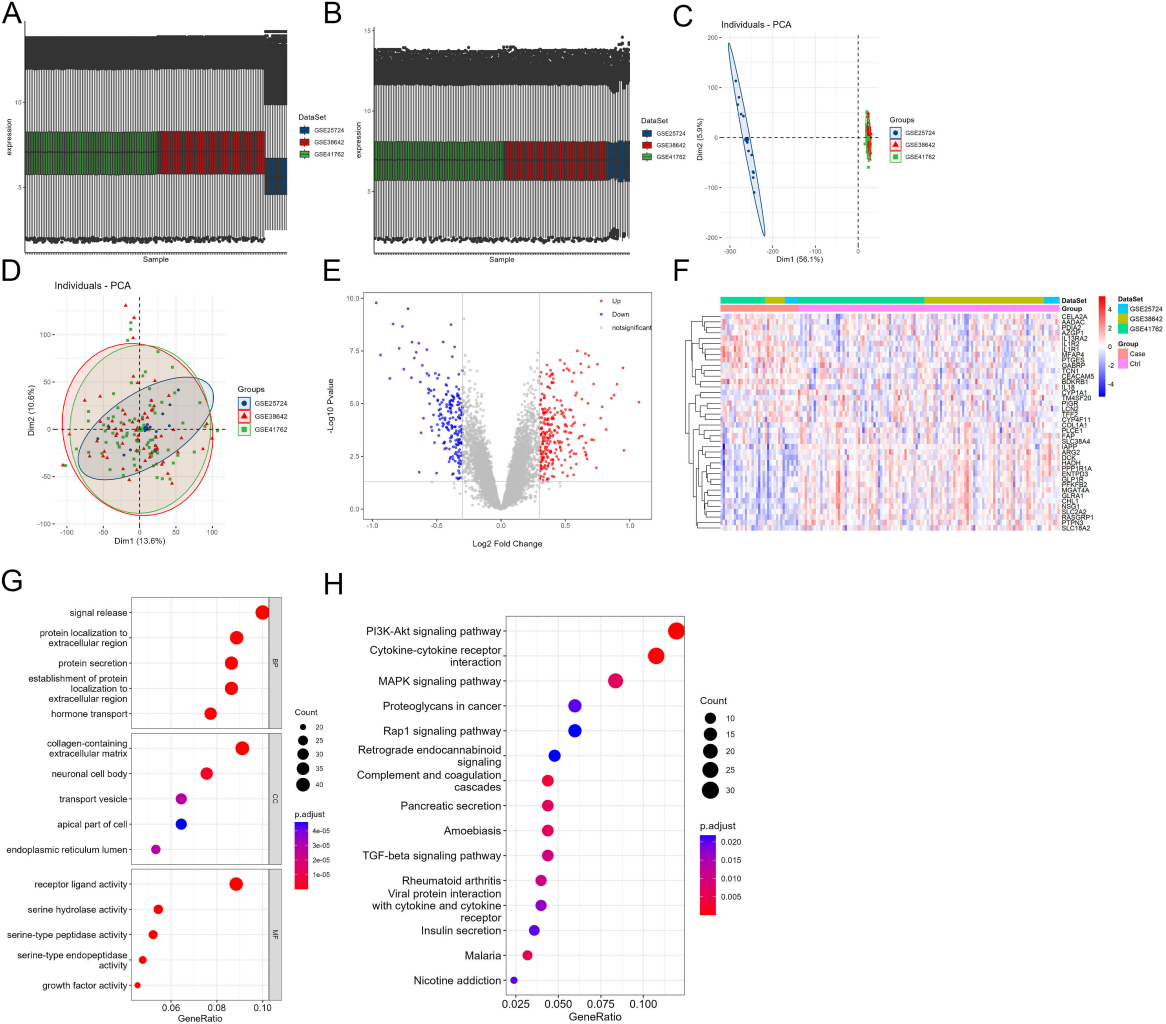
Identification of the DEGs in pancreatic islets from patients with or without T2DM. **(A, B)** The expressions of samples before **(A)** and after **(B)** batch effect removing. **(C, D)** PCA analysis of gene expression profiles in the merged dataset before **(C)** and after **(D)** batch effect removing. **(E)** Volcano plot of the DEGs in the merger dataset. Red meant upregulated, blue meant downregulated, and gray meant no significant difference. **(F)** Heatmap of the DEGs on the top 40 in the merger dataset. **(G, H)** GO **(G)** and KEGG **(H)** enrichment analysis of the DEGs.

In order to obtain the DEGs in pancreatic islets from patients with or without T2DM, we first merged the datasets of GSE41762, GSE38642, and GSE25724. A total of 35 samples with T2DM and 118 control samples were included. After removing the batch effect, the samples of different datasets that showed different clustering patterns (**Figures 1A, C**) would display the same clustering patterns (**Figures 1B, D**). A total of 458 DEGs were obtained using the “limma” package of R with the threshold values of |logFC| >0.3 and *p* < 0.05; among them, 255 DEGs were upregulated and 203 DEGs were downregulated (**Figure 1E**, **Supplementary Table S1**). The top 40 DEGs are shown in **Figure 1F**. Subsequently, GO and KEGG enrichment analyses were used to explore the function of DEGs. As shown in **Figure 1G** and **Supplementary Table S2**, signal release, protein localization to extracellular region, and protein secretion were enriched in the biological process. Collagen-containing extracellular matrix, neuronal cell body, and transport vesicle were enriched in cellular component. The results of molecular function analysis showed that receptor ligand activity, serine hydrolase activity, and serine-type peptidase activity were enriched. Furthermore, KEGG analysis revealed the DEGs were mainly associated with PI3K-Akt signaling pathway, cytokine–cytokine receptor interaction, and MAPK signaling pathway (**Figure 1H**, **Supplementary Table S3**).

### 3.2 Identification of the hub DEGs related to mitochondria in pancreatic islets from patients with or without T2DM

To explore the hub DEGs related to mitochondria in pancreatic islets from patients with or without T2DM, we picked out the mitochondrial genes from DEGs and nine mitochondria-related DEGs were obtained, including ATP citrate lyase (ACLY), 4-aminobutyrate aminotransferase (ABAT), interferon alpha inducible protein 27 (IFI27), arginase 2 (ARG2), dehydrogenase/reductase 2 (DHRS2), epoxide hydrolase 2 (EPHX2), alpha-methylacyl-CoA racemase (AMACR), hydroxyacyl-CoA dehydrogenase (HADH), and abhydrolase domain containing 10, depalmitoylase (ABHD10) (**Supplementary Figure S1A**). Their locations on chromosomes are shown in **Supplementary Figure S1B**. Then, the ssGSEA enrichment scores of mitochondria-related DEGs were analyzed using the “GSVA” package of R language. The results revealed that the ssGSEA enrichment scores of mitochondria-related DEGs were obviously lower in the samples with T2DM than those in the control samples (**Supplementary Figure S1C**).

**Figure 2:**
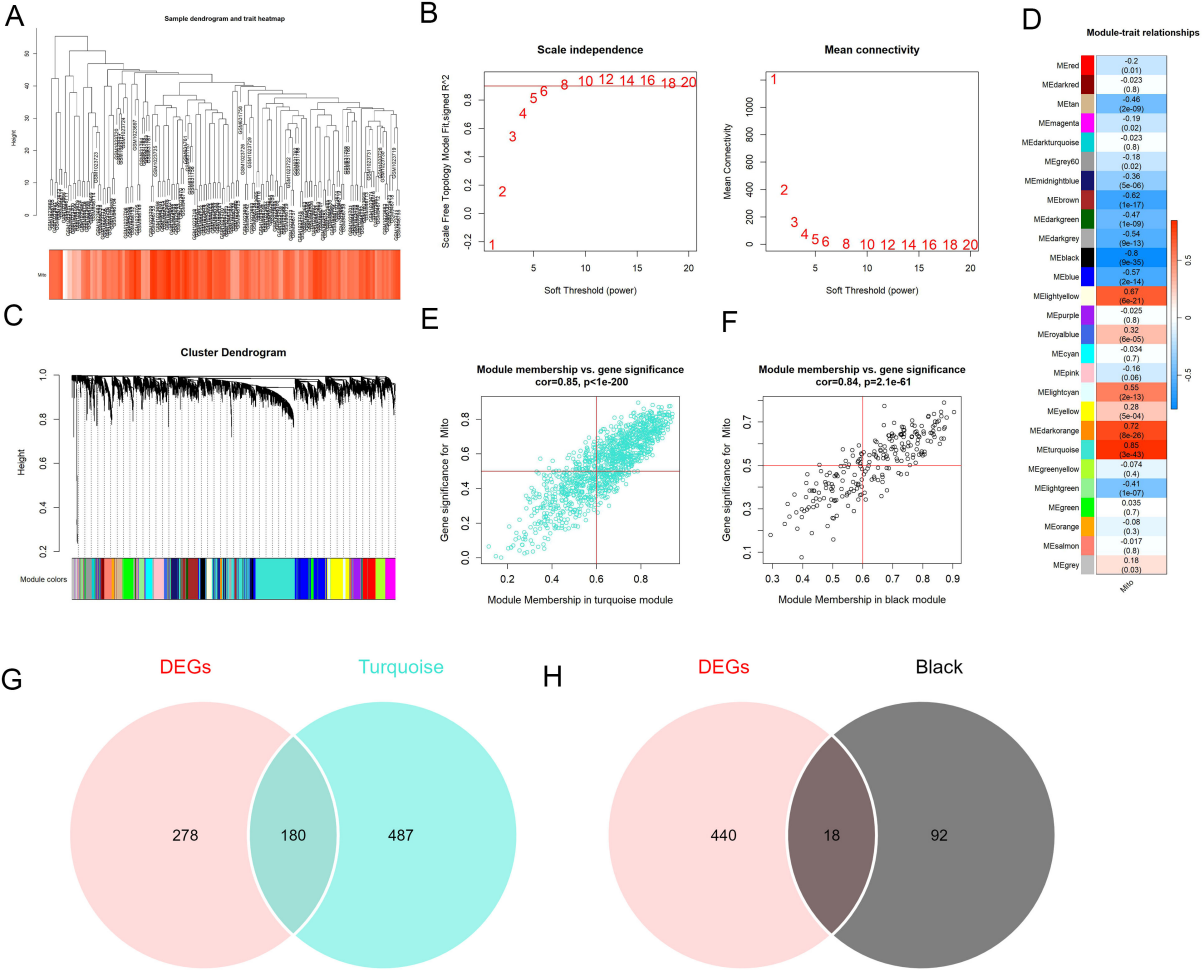
Identification of the hub DEGs related to mitochondria in pancreatic islets from patients with or without T2DM. **(A)** Clustering analysis of the samples specific to mitochondria in the merged dataset. **(B)** Analysis of scale-free topological networks and connectivity in different soft-threshold powers. **(C)** Cluster dendrogram of the gene expression modules specific to mitochondria. **(D)** The module–trait relationships with the nine mitochondria-related DEGs. **(E, F)** Correlation of the module membership in the turquoise module **(E)** and the black module **(F)** with the gene significance of the nine mitochondria-related DEGs. **(G, H)** The Venn diagrams of DEGs with genes in the turquoise module **(G)** or the black module **(H)**.

Subsequently, the gene expression modules specific to mitochondria in the merged dataset were established using “WGCNA” package based on the ssGSEA enrichment scores of the nine mitochondria-related DEGs. No outliers were found in the samples after clustering analysis (**Figure 2A**). Scale-free topological networks and connectivity were optimal when the soft threshold β was set to 6 in the PickSoftThreshold function (**Figure 2B**). A total of 26 gene modules with different color were obtained by hierarchical clustering (**Figure 2C**). Among these modules, turquoise module with R = 0.85 and black module with R = −0.8 had the strongest correlations with the nine mitochondria-related DEGs (**Figure 2D**). In addition, 667 hub genes and 110 hub genes were obtained in the turquoise module (**Figure 2E**, **Supplementary Table S4**) and black module (**Figure 2F**, **Supplementary Table S5**), respectively, by setting the |GS| > 0.5 and |MM |> 0.6. After intersecting the hub genes identified in the turquoise module and black module with the DEGs in the merged dataset, we finally obtained 180 hub DEGs in the turquoise module (**Figure 2G**) and 18 hub DEGs in the black module (**Figure 2H**). These 198 hub DEGs were combined for further analysis.

### 3.3 Identification of the mitochondria-related feature genes in T2DM

**Figure 3:**
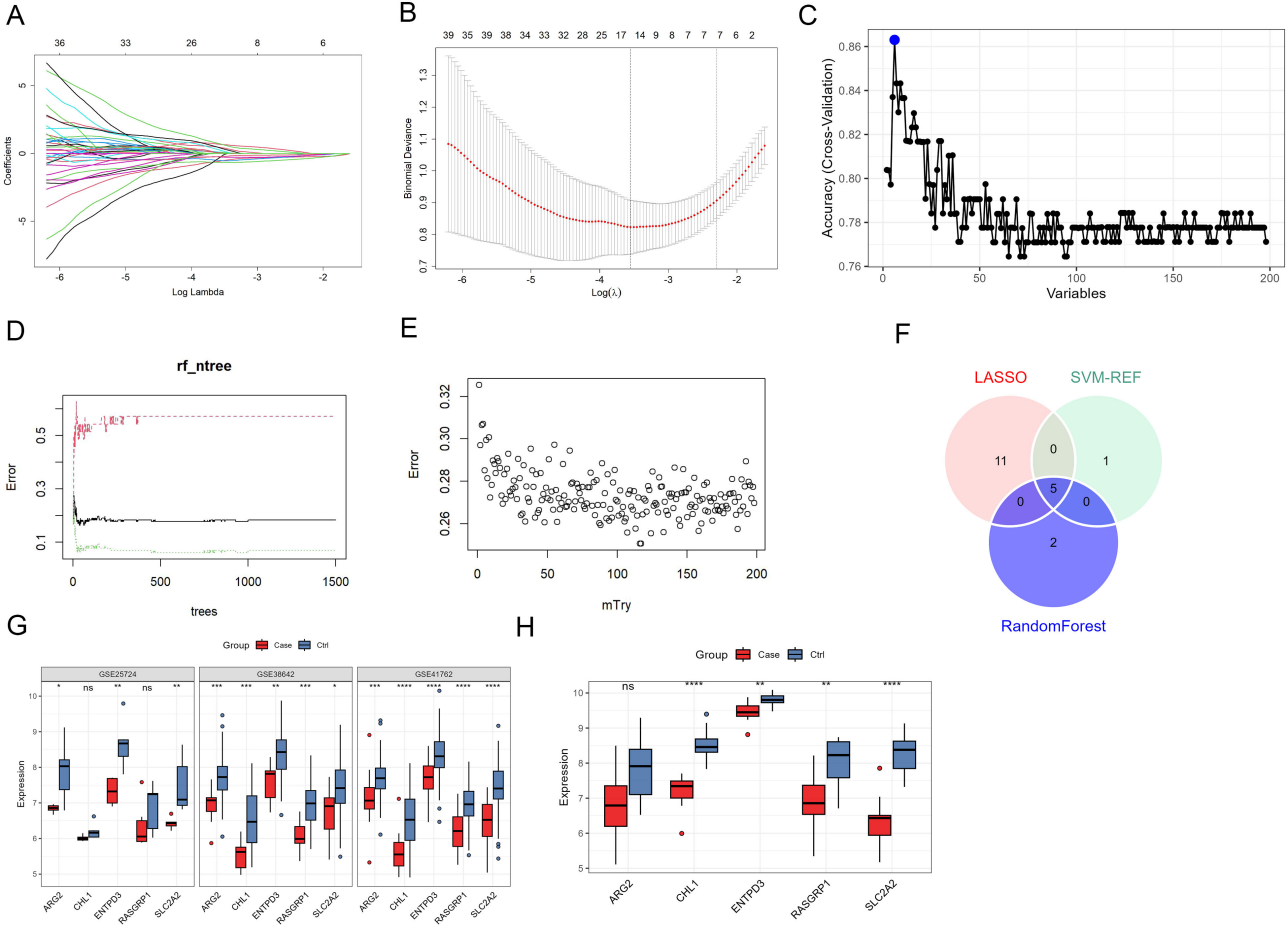
Identification of the mitochondria-related feature genes in T2DM. **(A, B)** The lambda **(A)** and γ **(B)** values screened by LASSO algorithm. **(C)** The accuracy of SVM-RFE model in different variables. **(D, E)** The error rate of RF model in different ntree **(D)** and mtry **(E)** values. **(F)** The Venn diagrams of feature genes obtained from LASSO, SVM-RFE, and RF models. **(G, H)** The expressions of SLC2A2, ENTPD3, ARG2, CHL1, and RASGRP1 in the datasets of GSE41762, GSE38642, GSE25724, and GSE20966. * represents *P*<0.05, ** represents *P*<0.01, *** represents *P*<0.001, and **** represents *P*<0.0001.

Next, we employed three machine learning algorithms, including SVM-RFE, LASSO, and RF to explore the mitochondria-related feature genes in the 198 hub DEGs. Based on the minimum lambda value, LASSO regression obtained 16 mitochondria-related feature genes with non-zero coefficients (**Figures 3A, B**). SVM-RFE model had the highest accuracy when the number of mitochondria-related feature genes was 6 (**Figure 3C**). The error rate of the RF model tended to be stable when the ntree value was >1,100 (**Figure 3D**), it would be the lowest when the mtry value was 116 (**Figure 3E**). Finally, seven genes with a Gini coefficient >1 were considered as mitochondria-related feature genes screened by RF. After intersecting the mitochondria-related feature genes identified by the three algorithms, five mitochondria-related feature genes were obtained, including solute carrier family 2 member 2 (SLC2A2), ectonucleoside triphosphate diphosphohydrolase 3 (ENTPD3), ARG2, cell adhesion molecule L1 like (CHL1), and RAS guanyl releasing protein 1 (RASGRP1) (**Figure 3F**). We further explored the expressions of these five mitochondria-related feature genes in individual dataset. The results showed all of the feature genes were lower in disease samples with T2DM than those in the control samples (**Figure 3G**). Their expressions were further validated by another dataset GSE20966, which contained the mRNA expression of beta-cells from pancreases of patients with or without T2DM (**Figure 3H**).

### 3.4 Assessment of the accuracies of the mitochondria-related feature genes in different datasets

**Figure 4:**
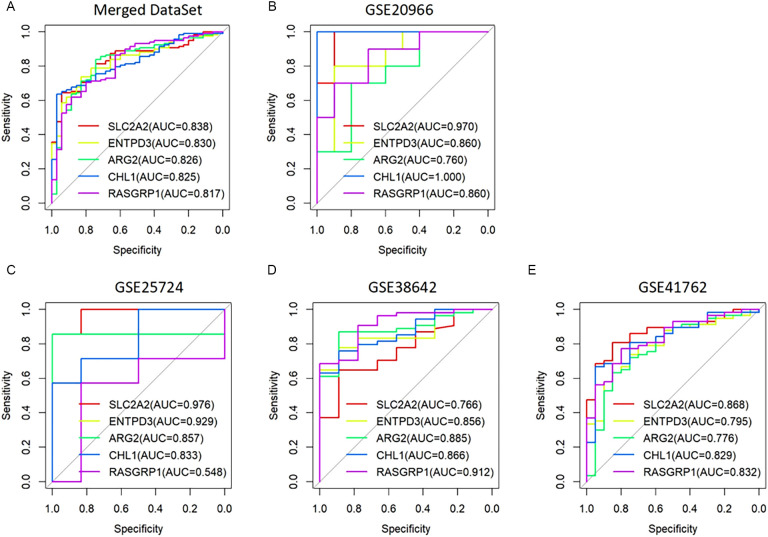
Assessment of the accuracies of the mitochondria-related feature genes in different datasets. **(A)** ROC curves of SLC2A2, ENTPD3, ARG2, CHL1, and RASGRP1 in the merged dataset. **(B)** ROC curves of SLC2A2, ENTPD3, ARG2, CHL1, and RASGRP1 in the validation set GSE20966. **(C–E)** ROC curves of SLC2A2, ENTPD3, ARG2, CHL1, and RASGRP1 in datasets of GSE41762, GSE38642, and GSE25724.

To assess the accuracies of the five mitochondria-related feature genes, ROC analysis was used to calculate the value of the area under the ROC curve (AUC). As shown in **Figure 4A**, the AUC values were 0.838 for SLC2A2, 0.830 for ENTPD3, 0.826 for ARG2, 0.825 for CHL1, and 0.817 for RASGRP1 in the merged dataset, demonstrating superior performance compared to the traditional Finnish Diabetes Risk Score (FINDRISC), which typically achieves AUROC values in the range of 0.70 to 0.78 (21, 22). Then, we further evaluated the accuracies of the five mitochondria-related feature genes in another dataset GSE20966. Their AUC values were 0.970 for SLC2A2, 0.860 for ENTPD3, 0.760 for ARG2, 1.000 for CHL1, and 0.860 for RASGRP1 (**Figure 4B**). In addition, the AUC values of the five mitochondria-related feature genes were also analyzed in datasets of GSE41762, GSE38642, and GSE25724. Their AUC values were 0.976 for SLC2A2, 0.929 for ENTPD3, 0.857 for ARG2, 0.833 for CHL1, and 0.548 for RASGRP1 in dataset GSE25724 (**Figure 4C**). AUC values were 0.766 for SLC2A2, 0.856 for ENTPD3, 0.885 for ARG2, 0.866 for CHL1, and 0.912 for RASGRP1 in dataset GSE38642 (**Figure 4D**). AUC values were 0.868 for SLC2A2, 0.795 for ENTPD3, 0.776 for ARG2, 0.829 for CHL1, and 0.832 for RASGRP1 in dataset GSE41762 (**Figure 4E**). The results revealed that the five mitochondria-related feature genes had high accuracies.

### 3.5 Clinical diagnostic performance of the mitochondria-related feature genes for type 2 diabetes mellitus

**Figure 5:**
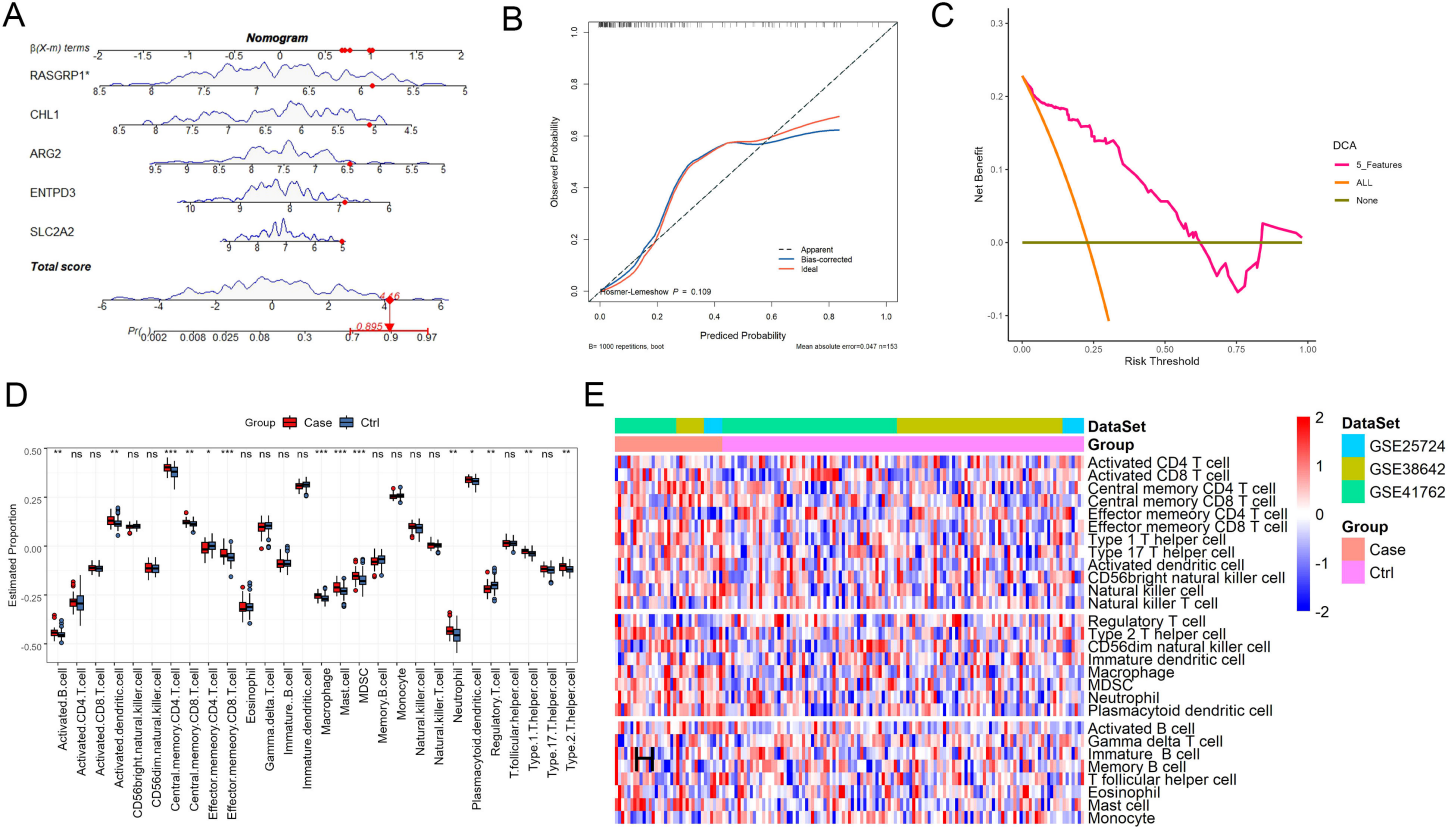
Clinical diagnostic performance of the mitochondria-related feature genes for type 2 diabetes mellitus. **(A)** The diagnostic nomogram established based on the mitochondria-related feature genes. **(B)** The calibration curves of the diagnostic nomogram. **(C)** The decision curves of the diagnostic nomogram. **(D)** The infiltrations of immune cells in the pancreatic islets of patients with T2DM compared with those in control individuals. **(E)** Heatmap of the distributions of immune cell subtypes in the datasets of GSE41762, GSE38642, and GSE25724. * represents *P*<0.05, ** represents *P*<0.01, and *** represents *P*<0.001.

To further evaluate the clinical diagnostic performance of the mitochondria-related feature genes for type 2 diabetes mellitus, the diagnostic nomogram was established by performing the “rms” package of R language based on the mitochondria-related feature genes (**Figure 5A**). The calibration curves indicated that the probability of T2DM had good agreement between prediction by the nomogram and actual observation in T2DM (**Figure 5B**). Furthermore, the DCA curves showed patients could achieve more net benefit for T2DM diagnosis by using the nomogram, which was established based on the mitochondria-related feature genes (**Figure 5C**).

### 3.6 Identification of the differentially infiltrated immune cells in pancreatic islets from patients with or without T2DM

The infiltration of immune cells plays an important role in the development of T2DM, we wondered whether the mitochondria-related feature genes would be related to the infiltration of immune cells. First, we analyzed the ssGSEA enrichment scores of 28 immune cell subtypes in the merged dataset. The results showed that 14 immune cell subtypes were differentially infiltrated into the pancreatic islets of patients with T2DM compared with those in control individuals, including activated B cell, activated dendritic cell, central memory CD4 T cell, central memory CD8 T cell, effector memory CD4 T cell, effector memory CD8 T cell, macrophage, mast cell, myeloid-derived suppressor cells (MDSC), neutrophil, plasmacytoid dendritic cell, regulatory T cell, type 1 T helper cell, and type 2 T helper cell (**Figure 5D**). The distributions of the immune cell subtypes in the datasets of GSE41762, GSE38642, and GSE25724 are shown in **Figure 5E**. Subsequently, we further explored the potential genes that might interact with the five mitochondria-related feature genes in the GeneMANIA database. A total of 20 candidate genes were found, such as contactin 6 (CNTN6), arginase 1 (ARG1), and ankyrin 1 (ANK1) (**Supplementary Figure S2A**). Pearson’s correlation analysis showed the five mitochondria-related feature genes were positive correlated with each other in the merged dataset (**Supplementary Figure S2B**). In addition, Pearson’s correlation analysis was used to calculate the correlations between feature genes and immune cells. The results showed that the five mitochondria-related feature genes had positive or negative correlations with most immune cells. Specifically, all of these genes had positive correlations with memory B cells, regulatory T cell, immature dendritic cell, and monocytes. They were negatively correlated with central memory CD4 T cell, effector memory CD8 T cell, and neutrophils (**Supplementary Figure S2C**). The correlations between RASGRP1 and activated B cell or regulatory T cell are displayed in **Supplementary Figures S2D, E**. These results indicated that the five mitochondria-related feature genes were involved in the infiltration of immune cells in T2DM.

### 3.7 Identification of candidate drugs that would target the mitochondria-related feature genes

To investigate the therapeutic potential of the five mitochondria-related feature genes, we further identified the candidate drugs that would target these genes by using the DGIdb database. We found that 2(S)-amino-6-boronohexanoic acid, difluoromethylornithine, and compound 9 could targeted ARG2. Metformin was the candidate drug for SCL2A2 (**Supplementary Figure S3**, **Supplementary Table S6**).

### 3.8 The expressions of the mitochondria-related feature genes in MIN6 cells treated with high glucose and PA

**Figure 6:**
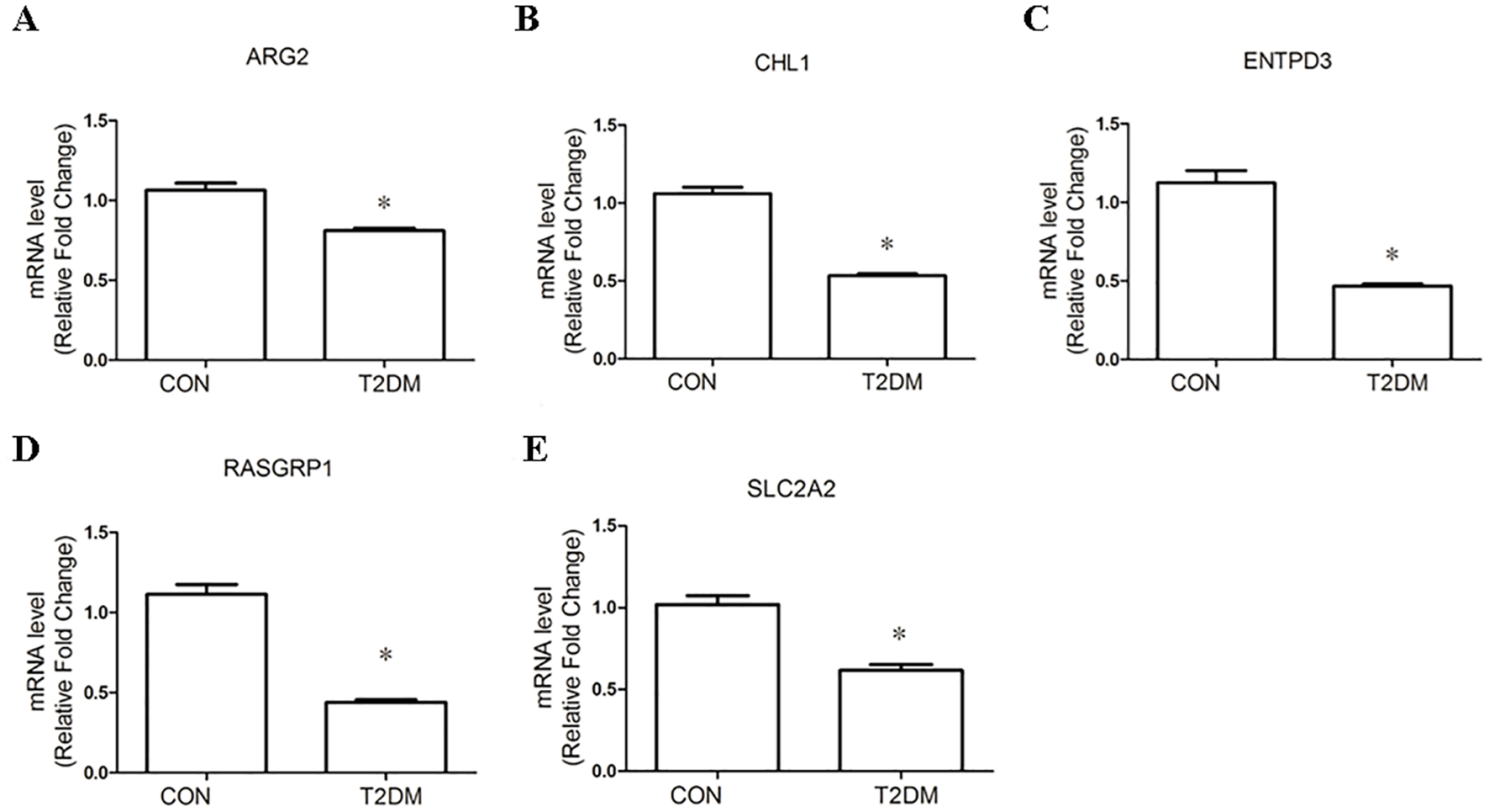
The expressions of the mitochondria-related feature genes in MIN6 cells treated with high glucose and PA. **(A–E)** The relative mRNA expressions of ARG2 **(A)**, CHL1 **(B)**, ENTPD3 **(C)**, RASGRP1 **(D)**, and SLC2A2 **(E)** in MIN6 cells treated with high glucose and PA or low glucose for 48 h GAPDH was used as internal reference. N=3, *p<0.05.

To further explore the expressions of the mitochondria-related feature genes in T2DM, we performed a glucose-lipid toxicity (GLT)-induced cellular T2DM model in MIN6 cells. The RT-PCR results showed that the mRNA levels of SLC2A2, ENTPD3, ARG2, CHL1, and RASGRP1 were significantly lower in MIN6 cells in the T2DM group than those in the control group (**Figures 6A–E**). These results revealed that the mitochondria-related feature genes identified by the bioinformatics method were changed in the cellular T2DM model, indicating that SLC2A2, ENTPD3, ARG2, CHL1, and RASGRP1 would important roles in the development of T2DM.

## 4 Discussion

T2DM is a metabolic disease with high blood glucose level and insulin resistance. An increasing number of individuals are living with T2DM (23). Mitochondria dysfunction plays critical role in the development of T2DM and immune cells infiltration (24, 25). However, whether there are any mitochondria-related genes that can predict the development of T2DM is unknown. In this study, we employed the machine learning methods to identify the mitochondria-related feature genes for T2DM prediction. A total of five mitochondria-related feature genes (SLC2A2, ENTPD3, ARG2, CHL1, and RASGRP1) were identified and confirmed to be decreased in the cell T2DM model. These genes were closely associated with immune cell infiltration. Overall, our study provided novel therapeutic targets for T2DM.

By using the machine learning methods, we identified five mitochondria-related feature genes for T2DM prediction, including SLC2A2, ENTPD3, ARG2, CHL1, and RASGRP1. SLC2A2, encoding the glucose transporter GLUT2, is critical for glucose sensing in pancreatic β cells and glucose uptake in the liver. GLUT2 deficiency in intestine attenuated glucose absorption and improved glucose tolerance (26). In addition, liver specific loss of GLUT2 also inhibited glucose uptake and impaired glucose-induced insulin secretion (27). Recent studies revealed that the SLC2A2 variation was related to T2DM treatment by influencing the effect of metformin on hemoglobin A1c reduction (28, 29). A bulk RNA-seq and single-cell RNA-seq analysis identified that ENTPD3 was a candidate target gene for T2DM (30). ARG2 was also considered as the feature gene of T2DM and related to immune response (31). In terms of mechanism, ARG2 activation was associated with ROS production and insulin resistance (32). Furthermore, CHL1 was reported to be decreased in islets of mice with T2DM. Deficiency of CHL1 would reduce insulin secretion via activating ERK/MAPK signaling pathway (33). In line with the previous studies, we revealed that SLC2A2, ENTPD3, ARG2, CHL1, and RASGRP1 could serve as the feature genes for T2DM prediction. Even though the underlying mechanisms were not fully understood, we hypothesized that these genes would influence the mitochondrial functions in T2DM because the five feature genes were obtained in the DEGs of T2DM based on the ssGSEA enrichment scores of mitochondria-related DEGs in T2DM.

The KEGG enrichment assay revealed that the DEGs of T2DM were primarily related to PI3K-Akt and MAPK signaling pathways. PI3K-Akt pathway was important for the signal transduction of insulin (34). However, the dysregulation of PI3K-Akt pathway would result in insulin resistance and the complications of T2DM (35). PI3K-AKT pathway could use as the target pathway for T2DM therapy. Resolvin D1 could improve the insulin response via activating PI3K-Akt pathway, then preventing the development of T2DM in mice (36). In addition, Irisin increased the secretion of insulin and alleviated insulin resistance by the activation of PI3K-AKT pathway (37). Phlorizin could activate PI3K-AKT signaling and attenuate the insulin resistance (38). Interestingly, our study found that one of the enriched pathways of DEGs in T2DM was PI3K-AKT signaling, indicating the DEGs identified in our study would partially work through PI3K-AKT signaling.

Immune cells played important role in the development of T2DM. Dendritic cells and Th17 cells were increased in the blood of patients with T2DM (39). Another study also found that the number of plasmacytoid dendritic cells were upregulated in the adipose tissue of patients with T2DM (40). Depletion of dendritic cells could attenuate the production of pro-inflammatory cytokines and improve the progression of T2DM (41). Furthermore, when the immune cells infiltrated into the injured tissue, they would secret the pro-inflammatory cytokines and induce ROS production, which ultimately led to insulin resistance (42–44). Consistent with these studies, our study revealed that all of the five mitochondria-related feature genes were negatively correlated with active dendritic cells and plasmacytoid dendritic cells. We also confirmed that the five mitochondria-related feature genes were downregulated in cellular T2DM model, so the increase in dendritic cells in T2DM might be associated with the decrease in the five mitochondria-related feature genes. However, the directly correlation and underlying mechanisms of the feature genes and dendritic cells infiltration need further investigation. In addition, even though the associations between the five mitochondria-related feature genes and immune cell infiltration in T2DM were not clear, their relationships in other diseases were reported. A negative association was observed between SLC2A2 expression and the extent of immune cell infiltration in hepatocellular carcinoma (45). ENTPD3 was negatively related to the infiltration of M1 macrophages, NK cells, and T cells in recurrent implantation failure patients (46). Hence, it was possible that the five mitochondria-related feature genes might affect the progression of T2DM disease by regulating immune cell infiltration.

Even though we explored the mitochondria-related feature genes of T2DM in three datasets and further validated them in another validation dataset, the sample size was still small; a larger sample size would make the conclusion more solid. In addition, we only confirmed the mRNA expressions of the five feature genes in the MIN6 cells treated with high glucose and PA; more experiments are necessary to further validate the functions of these genes in the development of T2DM. While our current study was primarily exploratory and focused on mechanistic insights, we recognized the need for future research to address scalability and industrial implementation. This could include collaborations with industry stakeholders to develop practical applications, develop diagnostic kits or assays targeting these genes, or explore small molecules or biologics that modulated the expression or activity of these genes.

## 5 Conclusion

In this study, we identified five mitochondria-related feature genes (SLC2A2, ENTPD3, ARG2, CHL1, and RASGRP1) that were significantly associated with the development of T2DM. Utilizing these genes, we developed a predictive model that demonstrated high diagnostic accuracy for T2DM, highlighting their potential as biomarkers for early detection and diagnosis. Furthermore, our analysis revealed a strong correlation between these genes and immune cell infiltration, suggesting their involvement in the immune-related pathogenesis of T2DM. Experimental validation in MIN6 cells exposed to high glucose and PA confirmed the altered expression of these genes under diabetic conditions. Collectively, these findings underscore the potential of SLC2A2, ENTPD3, ARG2, CHL1, and RASGRP1 as novel therapeutic targets for T2DM, offering new insights into the molecular mechanisms underlying the disease and paving the way for targeted interventions.

## Data Availability

The original contributions presented in the study are included in the article/**Supplementary Material**, further inquiries can be directed to the corresponding author/s.

## Supplementary material

Supplementary Figure 1 Identification of the mitochondria-related DEGs. **(A)**, The Venn diagram of DEGs obtained from merged dataset and the mitochondria-related genes. **(B)**, The locations of the 9 mitochondria-related DEGs on chromosomes. **(C)**, ssGSEA enrichment scores of mitochondria-related DEGs.

Supplementary Figure 2 The correlation of the feature genes and the infiltration of immune cells. **(A)**, The PPI network between feature genes and their potential targeted genes. **(B)**, The correlation between the feature genes. **(C)**, The correlation of the feature genes and the infiltration of immune cells. **(D, E)**, The correlations between RASGRP1 and activated B cell **(D)** or regulatory T cell **(E)**.

Supplementary Figure 3 Identification of candidate drugs which would target the mitochondria-related feature genes. The network of feature genes and the candidate targeted drugs. Rose red color represented feature genes. Green color represented targeted drugs.
