# Supplementary figures and images for: Identification of mitochondria-related feature genes for predicting type 2 diabetes mellitus using machine learning methods
Source: Front Endocrinol (Lausanne). 2025 Mar 27;16:1501159. doi: 10.3389/fendo.2025.1501159 (PMC11983445; doi:10.3389/fendo.2025.1501159)

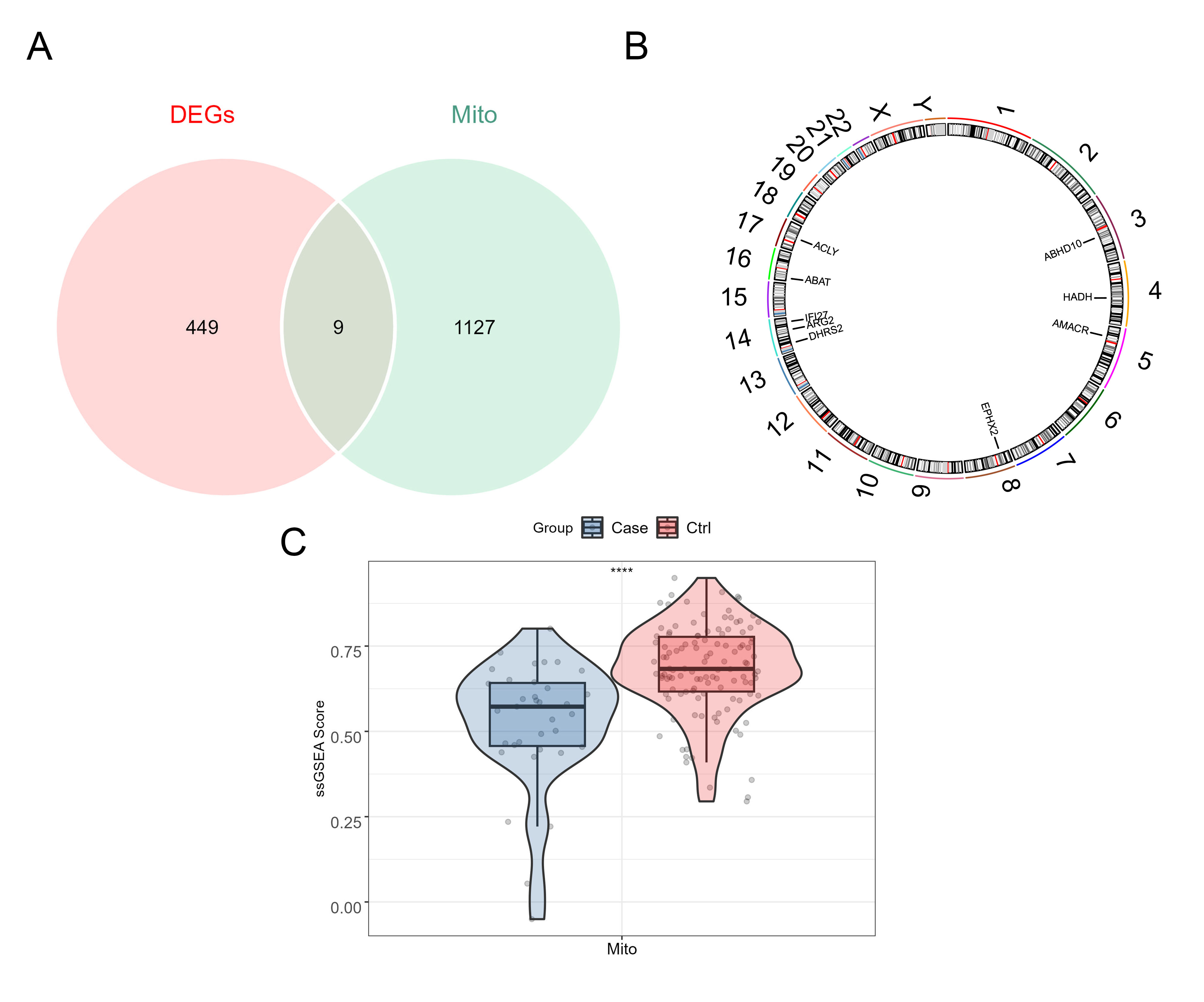

Supplement: Supplementary Figure 1 — Identification of the mitochondria-related DEGs. (A), The Venn diagram of DEGs obtained from merged dataset and the mitochondria-related genes. (B), The locations of the 9 mitochondria-related DEGs on chromosomes. (C), ssGSEA enrichment scores of mitochondria-related DEGs. [file Image1.jpeg]

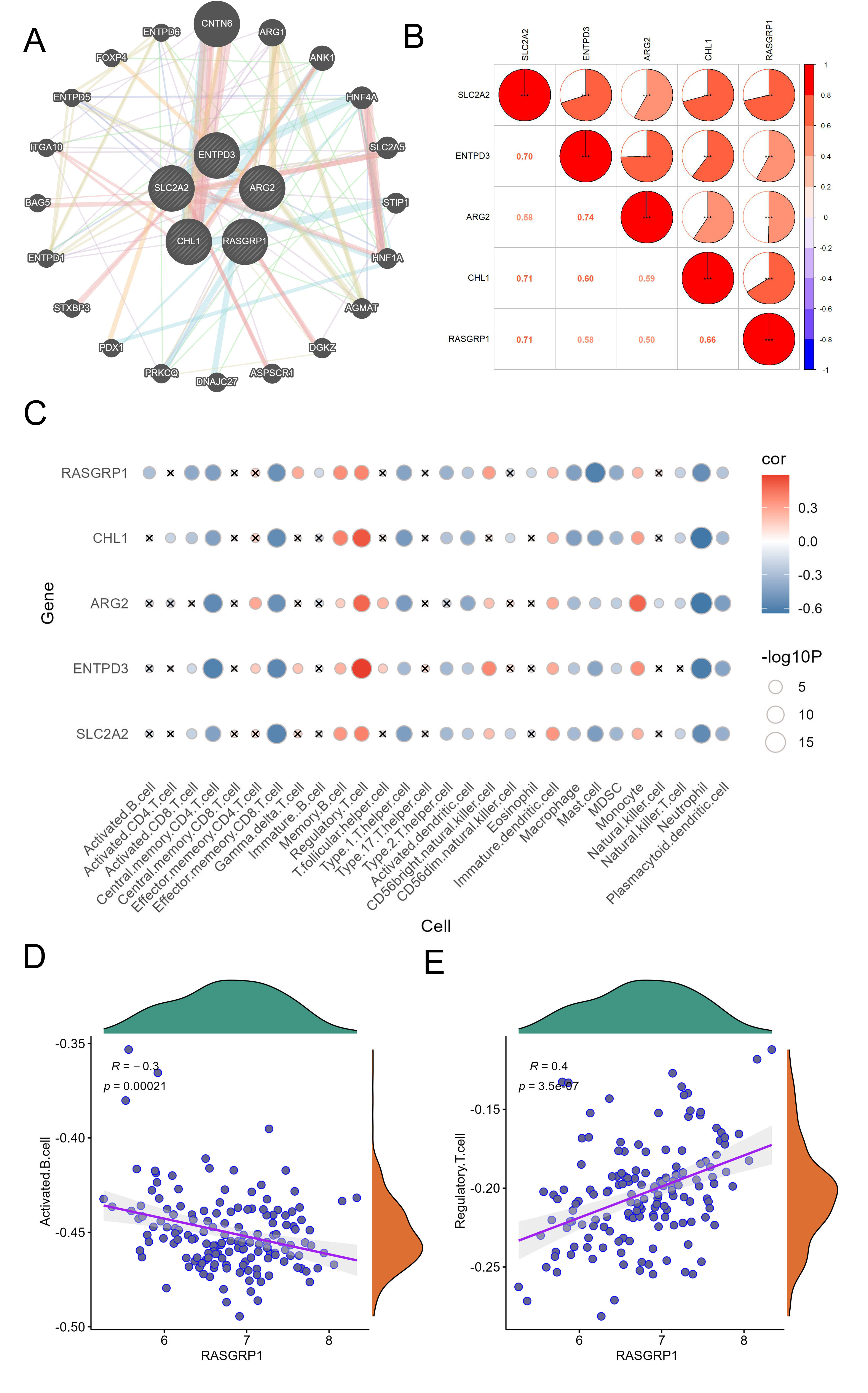

Supplement: Supplementary Figure 2 — The correlation of the feature genes and the infiltration of immune cells. (A), The PPI network between feature genes and their potential targeted genes. (B), The correlation between the feature genes. (C), The correlation of the feature genes and the infiltration of immune cells. (D, E), The correlations between RASGRP1 and activated B cell (D) or regulatory T cell (E). [file Image2.jpeg]

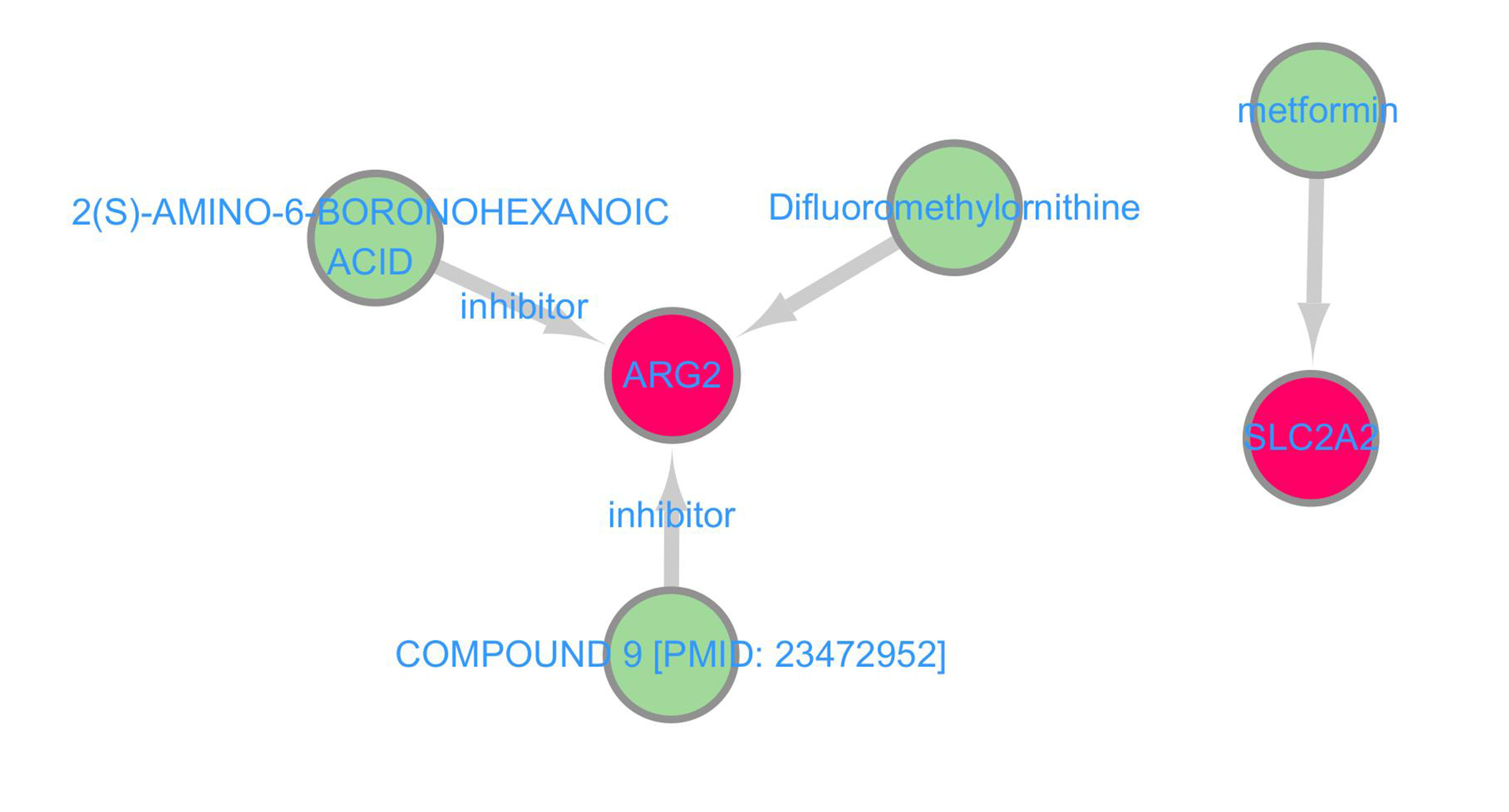

Supplement: Supplementary Figure 3 — Identification of candidate drugs which would target the mitochondria-related feature genes. The network of feature genes and the candidate targeted drugs. Rose red color represented feature genes. Green color represented targeted drugs. [file Image3.jpeg]
